# Assessing the multi-software robustness of radiomic biomarkers: a three-tool evaluation

**DOI:** 10.3389/fonc.2026.1764691

**Published:** 2026-06-26

**Authors:** Roberta Fusco, Giulia Festa, Mario Sansone, Sergio Venanzio Setola, Antonio Avallone, Francesco Izzo, Antonella Petrillo, Vincenza Granata

**Affiliations:** 1 Division of Radiology, Istituto Nazionale Tumori IRCCS Fondazione Pascale, IRCCS di Napoli, Naples, Italy; 2 Biomedical Engineering Faculty, Università degli Studi di Napoli Federico II, Naples, Italy; 3 Clinical Sperimental Abdominal Oncology Unit, Istituto Nazionale Tumori IRCCS Fondazione Pascale, IRCCS di Napoli, Naples, Italy; 4 Division of Epatobiliary Surgical Oncology, Istituto Nazionale Tumori IRCCS Fondazione Pascale, IRCCS di Napoli, Naples, Italy

**Keywords:** computed tomography, feature harmonization, oncology, radiomics, robustness

## Abstract

**Purpose:**

To assess the cross-software reproducibility of Computed Tomography (CT) radiomic features extracted using three widely adopted platforms (Siemens syngo.via Frontier, 3D Slicer/PyRadiomics, and mint Lesion) and to identify a subset of highly robust features suitable for multi-platform and multi-*center* radiomics applications.

**Methods:**

A retrospective cohort of 97 lesions (primary *color*ectal cancer*, color*ectal liver metastases, and hepatocellular carcinoma) who underwent contrast-enhanced Computed Tomography (CT) in the portal venous phase was *analyze*d. Semi-automatic 3D lesion segmentations were exported for radiomic extraction across the three platforms. Shared radiomic features *among* tools were harmonized and z-score normalized. Cross-platform similarity was assessed using distribution distance metrics, hierarchical clustering, and the Adjusted Rand Index (ARI). A novel Composite Robustness Index (CI) integrating Pearson correlation, Kolmogorov–Smirnov statistics, and mean fold-difference was developed to quantify feature-level reproducibility.

**Results:**

First-order intensity features and key GLCM descriptors (e.g., Correlation, Joint Average, Sum Entropy) demonstrated the highest cross-software stability, with nearly superimposable distributions and strong concordance in clustering structure. Siemens syngo.via Frontier and 3D Slicer/PyRadiomics showed the highest agreement (mean ARI *>*0.85)*, while* mint Lesion™—which lacks higher-order texture families—showed moderate deviations (mean ARI ≈ 0.70–0.75). High-order features, particularly GLDM and GLRLM metrics, exhibited substantial variability across platforms. The CI ranking enabled identification of a reproducible set of *“*highly reproducible features*, ”* including glcm_Correlation, firstorder_Mean, firstorder_RMS, firstorder_90Percentile, and shape axis-length descriptors.

**Conclusion:**

Despite intrinsic software differences, a consistent subset of radiomic features remains reproducible across heterogeneous extraction tools. The combined use of distribution analysis, hierarchical clustering, and the Composite Robustness Index offers a rigorous framework for evaluating cross-platform reliability. These findings support the feasibility of multi-tool radiomics and provide a validated feature set for harmonized quantitative imaging pipelines.

## 1 Introduction

Radiomics has emerged as a powerful quantitative imaging methodology capable of capturing spatial and intensity-based tissue characteristics beyond visual interpretation (1–8). Despite its increasing relevance in oncologic imaging, the lack of standardization in feature extraction pipelines remains one of the major barriers to clinical translation. Differences in software implementation, interpolation, discretization, and feature definitions can lead to substantial inconsistencies in radiomic values, even when using identical imaging data and segmentations (9–11).

To address these inconsistencies, the Image Biomarker Standardisation Initiative (IBSI) established standardized nomenclature and computational rules for radiomic feature calculation (9). Yet, several commonly used radiomic tools—both commercial and open-source—continue to exhibit subtle differences in their implementation of these standards, resulting in variability across software outputs (8). This issue is especially relevant in clinical environments where multiple quantitative imaging platforms coexist, such as syngo.via Frontier, 3D Slicer with PyRadiomics, and mint Lesion.

Radiomics from Mint Medical, which are used for research, radiology workflows, and oncologic decision support (9, 11).

Understanding the reproducibility of radiomic features across these platforms is essential for harmonization efforts, multi-center collaborations, and the development of robust imaging biomarkers (11–26).

The objective of this study was to systematically evaluate the cross-software reproducibility of Computed Tomography derived radiomic features extracted using Mint Medical, Siemens syngo.via Frontier, and 3D Slicer/PyRadiomics in a heterogeneous cohort of liver and colorectal malignancies. By comparing distribution similarity, hierarchical clustering patterns, and a composite reproducibility index, we identify robust features suitable for cross-platform radiomic applications.

## 2 Methods

### 2.1 Dataset characteristics

This study was conducted with the approval of the local Ethics Committee (Institutional Deliberation No. 323, March 5, 2023). The study cohort was derived from a multicancer population including patients with primary colorectal cancer (CRC), colorectal liver metastases (CRLM), and hepatocellular carcinoma (HCC).

A retrospective search of our institutional surgical registry was performed to identify consecutive patients with histologically confirmed CRC, CRLM, or HCC who had undergone preoperative contrast-enhanced CT. Eligibility required the availability of a diagnostic CT scan performed at our institution that included a portal venous phase acquired approximately 90 seconds after contrast administration, as well as at least one lesion that was clearly visible and suitable for semi-automatic three-dimensional segmentation. When multiple lesions met these criteria within the same patient, all of them were included to better capture real-world lesion variability and to enhance the assessment of feature reproducibility across radiomic platforms.

Exclusion criteria comprised severe motion or beam-hardening artifacts, absence of a portal venous phase, incomplete or corrupted DICOM metadata, and lesions deemed too small or insufficiently definable for accurate segmentation.

Applying these criteria, we included a total of 97 lesions from 71 patients, distributed as follows: 16 HCC, 45 primary CRC, and 36 CRC liver metastases. The median patient age was 62 years (range: 35–80 years).

### 2.2 CT imaging protocol

CT examinations were performed using either a 64-slice multidetector scanner (Optima 660, GE Healthcare, Chicago, IL, USA) or a dual-source spectral system (Somatom Drive, 256-slice, Siemens Healthineers, Erlangen, Germany). Acquisition para*meter*s typically ranged from 80 to 140 kVp and 100 to 470 mA, with reconstructed slice thickness between 2 and 2.5 mm. All patients received a non-ionic iodinated contrast agent administered at a flow rate of 3 mL/s via an automated injector (Empower CTA, EZ-EM Inc., New York, NY, USA). For radiomic analysis, only the portal venous phase images were considered, corresponding to a delay of approximately 90 seconds after intravenous contrast injection.

### 2.3 Image segmentation and radiomic feature extraction

Lesions were segmented using a semi-automated three-dimensional workflow previously reported in our manuscript (26), in which the operator provides an initial seed point or approximate contour that is subsequently refined by the algorithm into a full volumetric mask. All segmentations underwent systematic review and were manually adjusted when necessary, by two abdominal radiologists, each with over ten years of experience in oncologic imaging, to ensure anatomical precision and harmonization across cases. In patients with more than one eligible lesion, each lesion was segmented separately and analyzed as an independent observational unit. This approach reflects the study’s aim to evaluate cross-software radiomic feature robustness at the lesion level rather than to generate patient-level conclusions. The finalized 3D lesion masks were then used as the basis for radiomic feature extraction.

Radiomic features were extracted independently using three software platforms: syngo.via Frontier (Siemens Healthineers; Platform A), 3D Slicer with the PyRadiomics extension (version 5.6.2; Platform B), and mint Lesion™ (Mint Medical; Platform C).

Platform A — syngo.via Frontier (MM Radiomics Frontier, Siemens Healthineers): syngo.via Frontier is an integrated quantitative imaging environment that enables direct import of DICOM series, semi-automated 3D lesion segmentation, and computation of radiomic descriptors. The MM Radiomics Frontier module uses an embedded implementation of PyRadiomics compliant with the Image Biomarker Standardisation Initiative (IBSI) guidelines, generating first-order statistics, shape descriptors, and texture features. Feature outputs were exported in tabular format for external analysis.

Platform B — 3D Slicer with PyRadiomics: 3D Slicer is an open-source environment for advanced image visualization, segmentation, and quantitative analysis. Radiomic extraction was performed through the SlicerRadiomics extension, which relies on the PyRadiomics engine as its computational backend. The tool computes a broad set of IBSI-standardized first-order, shape, and texture features, offering fine control over preprocessing parameters such as resampling, discretization, and filtering.

Platform C — mint Lesion™ (Mint Medical GmbH, Heidelberg, Germany): mint Lesion™ is a commercial radiology workflow and quantitative analytics platform that performs automated calculation of radiomic biomarkers during routine clinical annotation. According to the vendor documentation, radiomic features in mint Lesion™ are implemented in full accordance with IBSI standards. The software provides first-order intensity features, histogram-based metrics, morphological descriptors, and second-order texture features derived exclusively from the gray-level co-occurrence matrix (GLCM). Features from higher-order texture families (GLRLM, GLSZM, GLDZM, NGTDM, NGLDM) are currently not implemented.

Across all three software platforms, radiomic feature extraction was performed using the finalized 3D lesion segmentations obtained from the portal venous phase CT images. To ensure that the resulting feature values were directly comparable, the exported tables from each platform underwent a dedicated harmonization process. This included alignment of feature names, verification of one-to-one correspondence across platforms, standardization of feature ordering, and normalization procedures described in the subsequent methodological sections. Regarding image-level preprocessing: for all three platforms, features were extracted from the original portal venous phase DICOM images without additional image-level intensity normalization or filtering. For Platforms A (syngo.via Frontier, MM Radiomics module) and B (3D Slicer version 5.6.2 with SlicerRadiomics/PyRadiomics), identical extraction settings were applied as described in our companion paper (26): all feature families were selected, default discretization settings were maintained, and wavelet filtering was activated with default parameters (decomposition level 1 and Coiflet 1 mother wavelet), yielding a total of 854 and 851 radiomic features respectively. For Platform C (mint Lesion™; Mint Medical GmbH, Heidelberg, Germany), the following configuration was applied: Fixed Bin Size (FBS) discretization with a bin width of 25 HU; GLCM computed over 3D average directions (3D:avg) with a distance of 1; no voxel resampling; no intensity threshold; no image filter (consistent with the absence of wavelet-derived features in this platform). These settings were directly confirmed from the platform interface used in this study. Regarding feature harmonization: following export from each platform, feature names were normalized and matched across platforms using a standardized string-distance algorithm. Since Platform C (mint Lesion™) does not implement wavelet-derived features, the common analytic dataset was restricted to “original” (non-wavelet) features. A total of 42 radiomic features were confirmed as common across all three platforms and constituted the analytic dataset for all subsequent cross-software comparisons. No intensity normalization prior to feature extraction was applied at the image level; z-score normalization was performed post-extraction on the tabular data as described in Section 2.4.

### 2.4 Radiomic datasets and pre-processing

Because numerical scaling differed across software packages, all features were first converted to numeric type and subsequently z-score normalized within each software dataset:

$z=\frac{x-\mu }{\sigma }$where *μ* and *σ* are the mean and standard deviation computed per feature within each software. This normalization ensures the distributions become directly comparable independently of the original intensity ranges.

To characterize similarity between feature distributions across software, five complementary metrics were computed for each tool: These metrics are defined in Equations 1–3.

- Pearson correlation distance

(1) $$d_{p}=\sqrt{2\left(1-r_{p}\right)}$$

- Spearman correlation distance

(2) $$d_{s}=\sqrt{2\left(1-r_{s}\right)}$$

- Euclidean distance
- Manhattan distance
- Cosine distance (computed using *philentropy*)

Distances were computed on Z-normalized data matrices, transposed so that each feature was treated as an individual variable vector.

**Figure 1:**
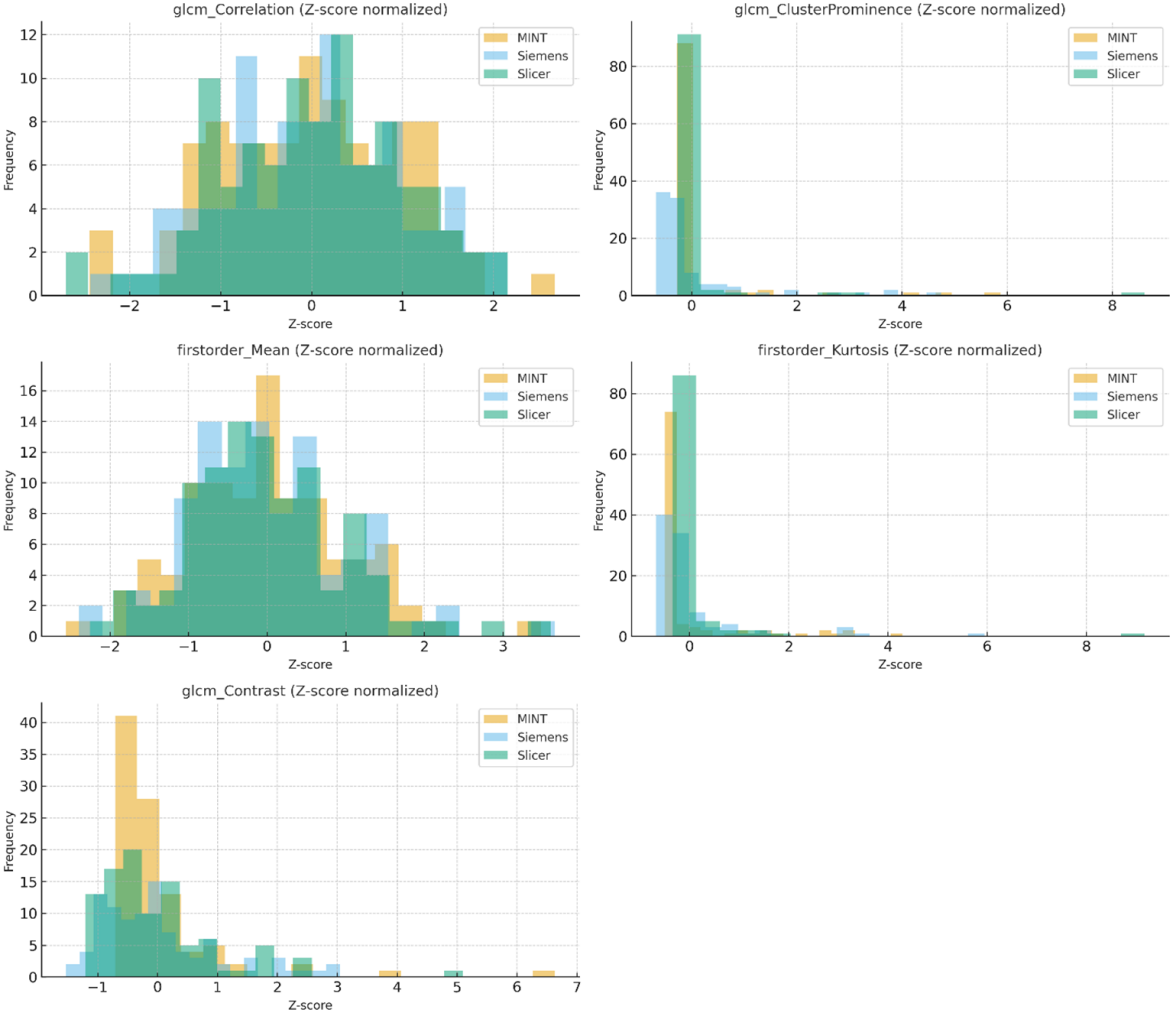
Example z-score–normalized distributions of representative radiomic features across the three software platforms: mint Lesion™ (Mint Medical GmbH, Heidelberg, Germany), syngo.via Frontier with MM Radiomics module (Siemens Healthineers, Erlangen, Germany), and 3D Slicer version 5.6.2 with SlicerRadiomics/PyRadiomics extension. Each panel shows the z-score-normalized distribution of a representative radiomic feature across all 97 lesions analyzed; overlapping curves indicate high cross-software agreement.

Representative examples of these normalized feature distributions are shown in **Figure 1**. For each software tool and each distance metric, hierarchical clustering was applied using the Ward.D2 linkage method. This procedure generates a feature-level dendrogram representing internal structural relationships among radiomic descriptors.

To determine the optimal number of clusters, the NbClust package was used with: Euclidean distance; minimum clusters = 2; maximum clusters = 12; linkage = Ward.D2. The optimal cluster count (*k*) was extracted from the majority vote of the NbClust criteria.

To quantify similarity between clustering structures produced by different software tools, the Adjusted Rand Index (ARI) was computed pairwise. For each pair, ARI values were computed for all combinations of distance metric and linkage method. ARI takes values from −1 to 1, where: 1 = identical clustering; 0 = chance-level agreement:<0 = systematic disagreement.

To quantify cross-software robustness of individual features, a Composite Index (CI) was computed for each feature and for each software pair:

- Pearson correlation (higher = more consistent)
- Kolmogorov–Smirnov statistic (lower = more consistent)
- Mean Fold-Difference (MFR) between distributions (lower = more consistent)

All components were z-scored and combined as:

(3) CI=z(r)−z(KS)−z(∣MFR∣)

A higher CI reflects stronger reproducibility of feature distributions between software tools. A final global CI_mean score was computed as the average CI across the three pairwise comparisons. Features were ranked in descending order of CI_mean. To ensure that the features entering the cross-software analysis already possessed adequate intra-tool measurement stability, a preliminary Intraclass Correlation Coefficient (ICC) analysis was conducted on each platform independently, prior to any cross-software comparison. Specifically, for each feature, the ICC (two-way model, absolute agreement, single rater; ICC(A, 1)) was computed across three repeated semi-automatic segmentations performed on the same lesions by the same operator (test–retest within platform). Features with ICC(A, 1)< 0.5 were excluded from subsequent cross-software analysis, retaining only those with at least moderate intra-tool reproducibility. This pre-filtering step is distinct from the cross-software CI analysis: the ICC here quantifies within-platform segmentation reproducibility, whereas the CI quantifies cross-platform distributional agreement on the pre-filtered feature set. The CI components (Pearson correlation, KS statistic, and mean fold-difference) were chosen to capture complementary aspects of feature agreement: linear association, distributional shape similarity, and magnitude consistency, respectively. Equal weighting after z-score normalization was adopted as a transparent and reproducible default. The CI is not intended to replace ICC but to provide an integrative cross-software summary that captures aspects of inter-platform robustness beyond what single-metric indices convey.

## 3 Results

After z-score normalization, all features showed comparable value ranges across all platforms. Several features, including GLCM Correlation, GLCM Cluster Prominence, and Histogram Mean, demonstrated nearly superimposable distributions across the three platforms, indicating high intrinsic stability in their numerical behavior. In contrast, features such as Intensity Kurtosis and NGTDM Contrast exhibited broader discrepancies in distribution shape and amplitude, reflecting increased sensitivity to software-specific implementation differences.

**Figure 2:**
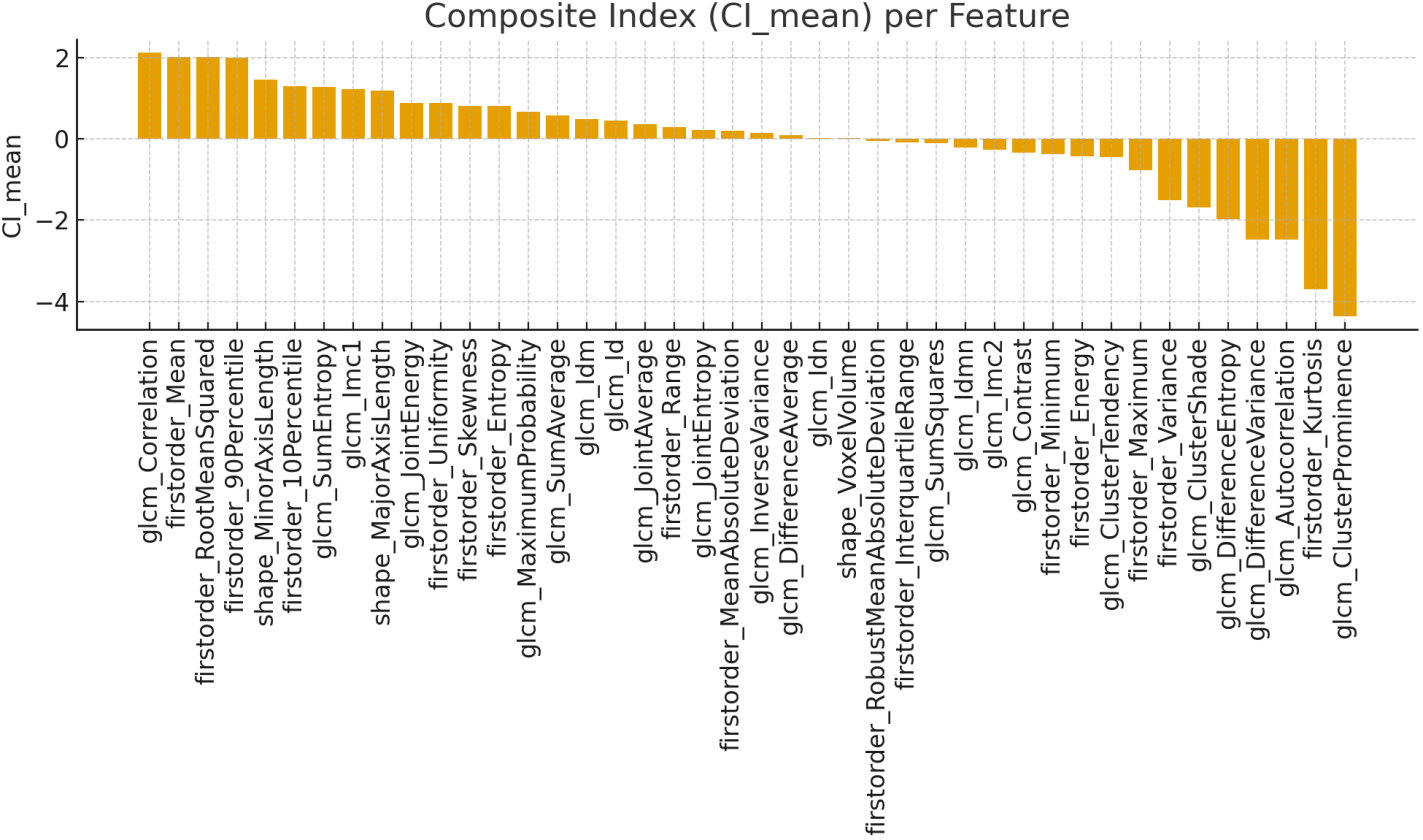
Composite Index mean (CI_mean) for radiomic features shared across mint Lesion™, syngo.via Frontier, and 3D Slicer/PyRadiomics. CI_mean is the average CI across all three pairwise comparisons. Higher CI_mean values denote greater cross-software reproducibility. The bar chart highlights a subset of highly stable features (right side) and features with reduced stability (left side).

Analysis of the CI revealed a wide spectrum of reproducibility across the shared features among tools. A subset exhibited high CI_mean values, reflecting strong cross-software consistency, while others demonstrated lower scores, suggesting susceptibility to variations in feature calculation or pre-processing across platforms (**Figure 2**).

Features with the highest reproducibility included: GLCM Correlation; GLCM Joint Average; Histogram Mean; Intensity RMS; GLSZM Small Area Emphasis.

These metrics showed excellent agreement in both distributional shape and inter-feature relationships.

In contrast, the lowest-ranked features—such as GLDM Dependence Entropy and GLRLM Short-Run High Gray-Level Emphasis—were among those most affected by software-specific implementation differences, particularly in high-order texture calculations.

The highest-ranked features on the Composite Index were predominantly GLCM-based texture metrics that demonstrated strong linear correlations across the three software packages. Several first-order intensity descriptors also showed minimal distributional divergence, confirming their robustness against software-specific implementation differences. In addition, selected GLRLM and GLSZM features maintained an almost identical distributional shape across platforms, supporting their stability.

By contrast, features with low CI_mean values generally exhibited large Kolmogorov–Smirnov discrepancies, inconsistencies in scaling after z-score normalization, or marked software-dependent sensitivity in histogram gradient calculations or dependence texture metrics. These patterns suggest greater susceptibility of these higher-order features to algorithmic variations in radiomic extraction pipelines.

To assess whether the structural relationships between radiomic features were preserved across extraction tools, we performed hierarchical clustering independently for MINT, Siemens Frontier, and 3D Slicer, using Ward.D2 linkage applied to Pearson distance matrices.

**Figure 3:**
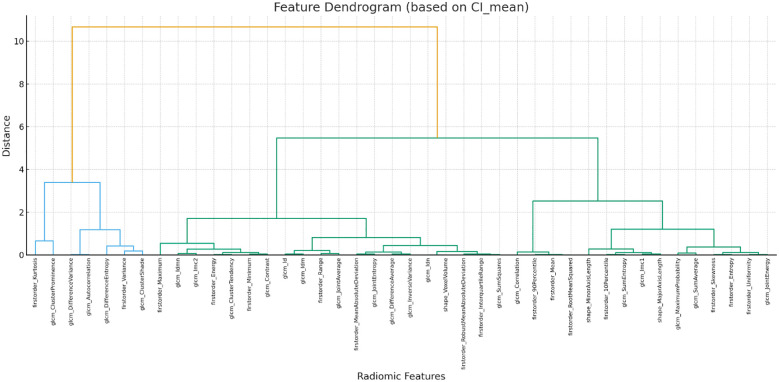
Hierarchical clustering dendrogram of the radiomic features computed across the three software platforms (MINT, Siemens Frontier, and 3D Slicer). Clustering was performed using the composite index (CI), which integrates Pearson correlation, Kolmogorov–Smirnov distance, and mean fractional difference across platforms. Features that cluster closely together demonstrate high cross-software agreement, whereas those branching at larger distances show greater variability. Colors indicate distinct feature clusters identified through hierarchical Ward linkage.

The resulting dendrograms (**Figure 3**) revealed a highly conserved organization of radiomic features across all three platforms. In particular, clusters comprising first-order, GLCM, GLRLM, GLDM, and GLSZM features appeared in nearly identical positions for Siemens and 3D Slicer, indicating strong concordance in their internal feature architecture.

In contrast, MINT showed modest deviations in the grouping of certain GLCM and GLDM metrics, suggesting subtle differences in its implementation of texture feature calculations. Nonetheless, the overall cluster topology remained broadly consistent, supporting the general reproducibility of feature–feature relationships across platforms.

The similarity among clustering solutions generated by the three software platforms was evaluated quantitatively using the Adjusted Rand Index (ARI). Across all combinations of distance metrics and linkage methods, Siemens Frontier and 3D Slicer demonstrated the greatest concordance, with mean ARI values consistently exceeding 0.85, indicating an almost identical hierarchical organization of radiomic features between these two tools.

In contrast, clustering similarity involving MINT was moderately lower, with mean ARI values ranging from approximately 0.70 to 0.75 when compared with either Siemens or Slicer. These findings suggest that MINT preserves the overall feature–feature relationship structure, but exhibits subtle deviations relative to the other platforms.

**Figure 4:**
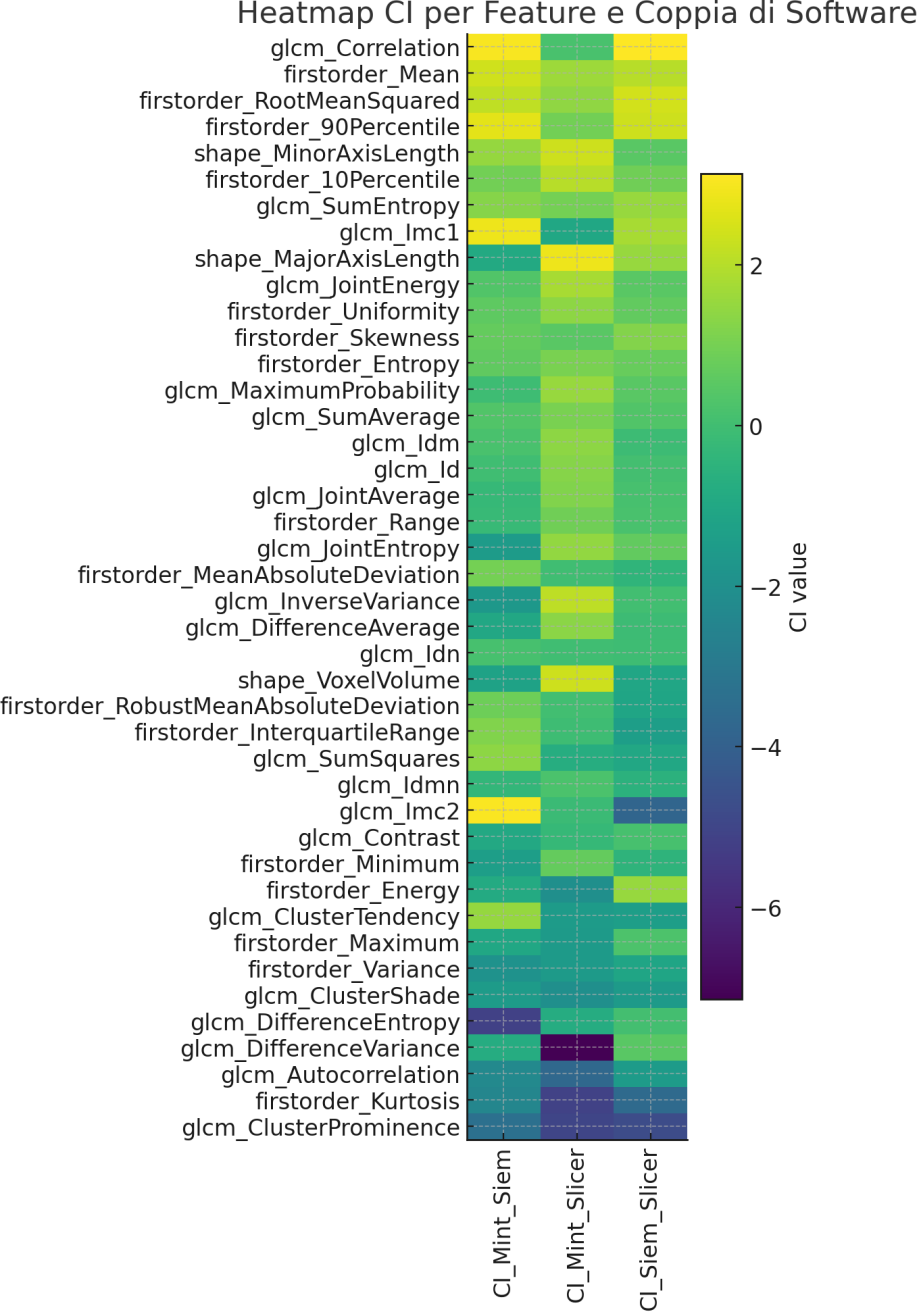
Heatmap showing the Composite Index (CI) values for each radiomic feature across the three software pairwise comparisons: mint Lesion™ vs syngo.via Frontier, mint Lesion™ vs 3D Slicer/PyRadiomics, and syngo.via Frontier vs 3D Slicer/PyRadiomics. Higher CI values (yellow) indicate strong cross-software agreement for a given feature, whereas lower CI values (blue–purple) denote reduced reproducibility and larger discrepancies across platforms. The heatmap highlights substantial stability for certain first-order and GLCM features, while others—particularly higher-order texture metrics—exhibit notable cross-software variability.

The complete set of pairwise ARI comparisons is presented in **Figure 4**, highlighting the degree of structural alignment among the three radiomic extraction pipelines.

Based on the clustering structure and CI mean values, a subset of “highly reproducible” features was identified as the most stable across the three extraction tools. Across all cluster partitions, at least one high-stability feature emerged from each radiomic class. The list of highly reproducible features is reported in **Table 1**.

**Table 1:** Final set of highest-robustness radiomic features (“highly reproducible features”).

Rank	Feature name	CI_mean
1	glcm_Correlation	2.122
2	firstorder_Mean	2.017
3	firstorder_RootMeanSquared	2.017
4	firstorder_90Percentile	1.997
5	shape_MinorAxisLength	1.453
6	firstorder_10Percentile	1.283
7	glcm_SumEntropy	1.265
8	glcm_Imc1	1.219
9	shape_MajorAxisLength	1.177
10	glcm_JointEnergy	0.885

## 4 Discussion

This study highlights meaningful differences in radiomic feature behavior across the three software platforms, emphasizing that even IBSI-compliant tools may implement certain processing steps differently. Among the evaluated platforms, Siemens Frontier and 3D Slicer/PyRadiomics exhibited a high degree of concordance, which is consistent with prior work showing that tools sharing similar feature definitions and discretization frameworks tend to produce comparable outputs (10, 14, 17). Mint Lesion Radiomics from Mint Medical also demonstrated strong agreement for a substantial portion of features, particularly first-order descriptors and several GLCM metrics. The differences likely stem from distinct preprocessing philosophies, including gray-level handling, voxel interpolation strategies, and image-specific normalization procedures, which are known to modulate radiomic measurements (18, 24).

Importantly, the composite robustness index and clustering analyses revealed feature families that were highly stable across all three software ecosystems. First-order intensity features (e.g., Mean, RMS, Percentiles) and key GLCM descriptors (e.g., Correlation, Joint Average, Sum Entropy) consistently demonstrated strong reproducibility. These findings echo earlier work identifying these groups as among the most reliable features in radiomics (25–31). Differences observed in NGTDM, GLDM, and certain GLRLM metrics align with evidence that higher-order features are naturally more sensitive to imaging parameters, reconstruction algorithms, and tool-specific discretization approaches (18, 24, 25). Thus, the observed variability should be interpreted as reflecting expected algorithmic diversity, not software inconsistency.

Our results are also aligned with our group’s previous multi-lesion robustness analysis (26) which demonstrated that carefully selected, stable radiomic features can maintain predictable behavior across different segmentation and acquisition environments. The present study expands this perspective by showing that cross-software agreement is achievable even when tools have heterogeneous internal architectures, provided that feature selection focuses on robust families.

Collectively, these findings reinforce the feasibility of multi-platform radiomics, highlight the importance of standardized preprocessing, and provide a concise list of “highly reproducible features” suitable for harmonized pipelines. Rather than favoring one tool over another, our results demonstrate that each platform contributes valuable radiomic information, and that the key to reproducibility lies in identifying feature classes that consistently withstand software-related variability.

Our results also align with the broader evidence emerging from singe and multicenter standardization studies (26, 32–41). That work demonstrated that substantial discrepancies persist between radiomic software packages even when using digital reference objects specifically designed to eliminate biological variability. The authors reported that differences in feature definitions, interpolation schemes, gray-level handling, and discretization strategies contributed to a lack of full cross-platform equivalence, highlighting the need for harmonized computational pipelines. Consistent with those findings, our tri-software analysis revealed that a non-negligible proportion of radiomic features—particularly higher-order texture descriptors—remains sensitive to software-specific implementations despite declared IBSI adherence. However, by combining distributional analysis, hierarchical clustering, and a novel Composite Robustness Index, our study adds granularity beyond the multicenter digital-object comparison, demonstrating that certain first-order and GLCM features retain strong reproducibility even across heterogeneous clinical software environments.

Furthermore, while the cited multicenter study emphasizes the challenges of achieving full computational standardization, our work contributes a practical outcome by identifying a set of robust “highly reproducible features” that remain stable despite cross-software variability. This supports the feasibility of constructing harmonized radiomic pipelines, provided that feature selection is informed by reproducibility evidence.

Several limitations of this study should be acknowledged. First, the dataset is derived from a single institution and focuses exclusively on portal venous phase CT acquisitions, which may limit the generalizability of the identified reproducible feature set to other imaging phases, modalities, or disease settings. Second, because patients with multiple eligible lesions contributed more than one observation to the analysis, the assumption of full statistical independence between lesions may not hold. This design choice was deliberate—it allowed us to capture real-world lesion-level variability and increase the sample size for feature reproducibility assessment—but intra-patient correlation could potentially inflate the precision of some estimates. Future studies should consider mixed-effects models or generalized estimating equations to formally account for lesion clustering within patients. Third, conclusions regarding cross-software reproducibility are necessarily conditional on the specific software versions and default settings used; updates to platform implementations may alter reproducibility profiles.

Moreover, future work should focus on harmonization techniques—such as ComBat, phantom-based calibration, and unified discretization strategies—to further minimize residual discrepancies and support cross-center radiomic reproducibility.

## 5 Conclusions

This tri-software evaluation shows that, despite differences in preprocessing and feature implementation, a core group of CT radiomic features—mainly first-order metrics and key GLCM descriptors—remains highly reproducible across Siemens syngo.via Frontier, 3D Slicer/PyRadiomics, and mint Lesion™. Specifically, the features with the highest CI_mean scores (i.e., highest cross-software distributional agreement across all three pairwise comparisons, combined with prior intra-tool ICC(A, 1) > 0.5) included: glcm_Correlation, glcm_JointAverage, glcm_SumEntropy, firstorder_Mean, firstorder_RMS, firstorder_90Percentile, shape_MajorAxisLength, and shape_MinorAxisLength. Higher-order texture features demonstrated greater variability, confirming their sensitivity to software-specific algorithms.

By combining distribution analyses, hierarchical clustering, and a Composite Robustness Index, this study provides a practical framework for assessing cross-platform consistency and identifies a reliable subset of “highly reproducible features” suitable for multi-center and multi-tool radiomic pipelines. These findings support the feasibility of harmonized radiomics workflows and underscore the importance of standardized preprocessing in quantitative imaging research.

## Data Availability

The original contributions presented in this study are included in the article and the extracted harmonized Radiomic features are available on https://zenodo.org/records/20593585.
